# Efficacy of platelet-rich plasma injection with percutaneous endoscopic lumbar discectomy for lumbar disc herniation: a systematic review and meta-analysis

**DOI:** 10.3389/fphar.2025.1622974

**Published:** 2025-09-03

**Authors:** Yu Zhang, Jidong Ju, Jinchun Wu

**Affiliations:** Department of Orthopaedics, Jingjiang People’s Hospital Affiliated to Yangzhou University, Taizhou, Jiangsu, China

**Keywords:** percutaneous endoscopic lumbar discectomy, platelet-rich plasma injection, lumbar disc herniation, efficacy, meta-analysis

## Abstract

**Objective:**

Our aim was to compare the efficacy of percutaneous endoscopic lumbar discectomy (PELD) with or without platelet-rich plasma (PRP) injections for the treatment of lumbar disc herniation (LDH).

**Methods:**

Literature was searched in six databases from inception to 2 May 2025. Meta-analysis was performed using stata17 software.

**Results:**

Eight studies were included, comprising a total of 717 patients (349 in the treatment group and 368 in the control group). At the final follow-up, the treatment group significantly provided lower visual analogue scale, intervertebral disc protrusion, recurrence rates, Oswestry disability index and higher intervertebral disc height, Japanese Orthopaedic Association scores, spinal canal cross-sectional area and ratio value of disc grey scale, compared to the control group (*p* < 0.05).

**Conclusion:**

Our findings indicate that for the treatment of LDH, PELD combined with PRP injection markedly enhances clinical efficacy while reducing recurrence rates compared to PELD alone. Moving forward, larger multicenter randomized controlled trials with extended follow-up periods are necessary to confirm the long-term efficacy and safety of this combination therapy.

**Systematic Review Registration:**

Identifier CRD420251044871.

## Introduction

Lumbar disc herniation (LDH) was a widespread and prevalent spine disorder among medical clinics, which seriously affected people’s normal work and life, and imposed a heavy burden on global health and socio-economics (Kong et al., 2020; Chen et al., 2024; Yang et al., 2024). LDH was mainly manifested as nerve root pains in the lower legs, sensory disturbances of skin around the distribution of the nerve roots, and limitation of limb movement (Huang et al., 2025; Jin et al., 2025). Effective treatments for LDH mainly included conservative and surgical treatments. For patients with incipient LDH, conservative treatments such as medication and physical function exercises were often preferred (Du et al., 2025; Brotis et al., 2025a; Brotis et al., 2025b). 90% of LDH patients could have their symptoms relieved after conservative treatments. However, surgical intervention was required when conservative treatment was ineffective or when neurological function was further aggravated (Mendieta et al., 2025; Wang et al., 2025).

Surgical treatment included minimally invasive and traditional open surgery. Conventional open operation was the classical procedure in treating LDH. However, it required extensive stripping of paraspinal muscle and large-scale resection of vertebral plate and facet joints, and resulted in complications such as spinal instability and chronic pain after surgery (Berg et al., 2024). Recently, thanks to the advancement of minimally invasive technology, spinal endoscopy had gradually become the mainstream clinical treatment of lumbar degenerative diseases for the merits of low trauma, fast rehabilitation and short hospital stay (Xiao et al., 2025; Ke et al., 2025; Cai et al., 2025). At present, percutaneous endoscopic lumbar discectomy (PELD) had been widespread applied for LDH management and obtained satisfactory efficacy. Proper resection of the intervertebral disc could alleviate the pains, but it would inevitably damage the structural integrity of discs and aggravate disc degeneration, and at the same time, the damaged annulus fibrosus would increase the chance of recurrence (Li and Wang, 2025; Zhao et al., 2025). Neither conservative nor surgical treatment could fundamentally address the underlying disc degeneration process in LDH. As a consequence, the enhancement of intervertebral disc reconstruction following discectomy was a key issue to improve the operative prognosis. Developments in regenerative medicine promoted the progress of experiments intended to restore and reconstruct healthy intervertebral discs, in particular growth factor therapies, cellular therapies and genetic therapies (Haro et al., 2025; Anitua et al., 2025).

As a biological therapy with good biosafety, simple preparation and easy source, platelet-rich plasma (PRP) had been shown to promote intervertebral disc cell proliferation and extracellular matrix synthesis, downregulate the expression of inflammatory factors, and inhibit apoptosis, which was expected to slow down or even reverse the pathological process of intervertebral disc degeneration (Lian et al., 2025; Zhang et al., 2024; Tao et al., 2024). Currently, PRP was widely used in the treatment of lumbar disc diseases and could achieve satisfactory efficacy (Kawabata et al., 2024; Singh et al., 2023). Minimally invasive spinal endoscopic removal of herniated intervertebral tissues could alleviate the symptoms of LDH patients, and PRP promoted the repair and regeneration of intervertebral disc tissues and restored the biomechanical function of the intervertebral discs, and the combination of the two techniques could play the effect of “1 + 1 > 2”.

PRP injection for treating low back pain *in vitro* had achieved encouraging outcomes. However, there was a paucity of evidence regarding the efficacy and security of PELD combined with PRP in treating LDH. The aim of this meta-analysis was to comprehensively evaluate the effectiveness and security of intraoperative PRP injections during PELD and to determine whether PRP injections might achieve favorable outcomes, enhance disc remodeling and reduce recurrence.

## Methods

This review was carried out according to the Preferred Reporting Items for Systematic Reviews and Meta-Analyses (PRISMA) statement (Page et al., 2021).

### Eligibility

#### Inclusion criteria

Participants: patients with a clinically and radiologically confirmed diagnosis of LDH requiring surgery.

Intervention: PELD combined with PRP as the treatment group.

Comparison: PELD only as the control group.

Outcomes: visual analogue score (VAS) for back pain and leg pain, intervertebral disc height (IDH), intervertebral disc protrusion (IDP), Oswestry disability index (ODI), Japanese Orthopaedic Association (JOA) score, spinal canal cross-sectional area (SCSA), recurrence, ratio value of disc grey scales (RVG). Studies included at least one of the above outcomes. The definitions and measurements of the outcome indicators were detailed in the Supplementary File 1.

Study design: observational studies, randomized controlled trials. Follow-up time was at least 1 year.

#### Exclusion criteria

1. Researches including patients with other lumbar spine conditions (e.g., fractures, infections, tumors) or with documented lumbar spine surgery.
2. Duplicate reports, case reports, reviews, meta-analyses, animal studies, biomechanical studies, letters, and conference abstracts.
3. Incomplete or unanalyzable raw information.

### Search strategy

A comprehensive literature search was carried out in 6 databases such as PubMed, Embase, Web of Science, Cochrane Library, Wanfang and China National Knowledge Infrastructure from the beginning of databases to 2 May 2025. The search formula was as follows (platelet rich plasma) AND (discectomy). Only English and Chinese publications were included.

### Data extraction

Two investigators separately extracted the following data from these eligible papers.

1. Study information: author name, publication year, country and study type.
2. Baseline characteristics: sample size, age, length of observation.
3. Endpoints to be investigated.

### Quality evaluation

Newcastle-Ottawa Scale (NOS) was used to determine the quality of observational researches. The total score was 9, with ≥6 considered high quality.

### Statistical analyses

Meta-analyses were performed using Stata 17.0. Heterogeneity of included researches was determined via I^2^ statistic. Effect sizes were pooled using a random-effects model when I^2^ >50% indicated significant heterogeneity between included studies. For continuous outcomes, effect sizes were expressed as weighted mean difference (WMD) and 95% confidence intervals (CI). For dichotomous outcomes, effect sizes were estimated as odds ratios (ORs) and 95% CIs. Sensitivity analyses were performed by stepwise deletion of each individual trial to determine the robustness of the combined results. Egger test was employed to objectively investigate publication bias. *P* < 0.05 was used to demonstrate statistical significance.

## Results

### Search process and results

**FIGURE 1:**
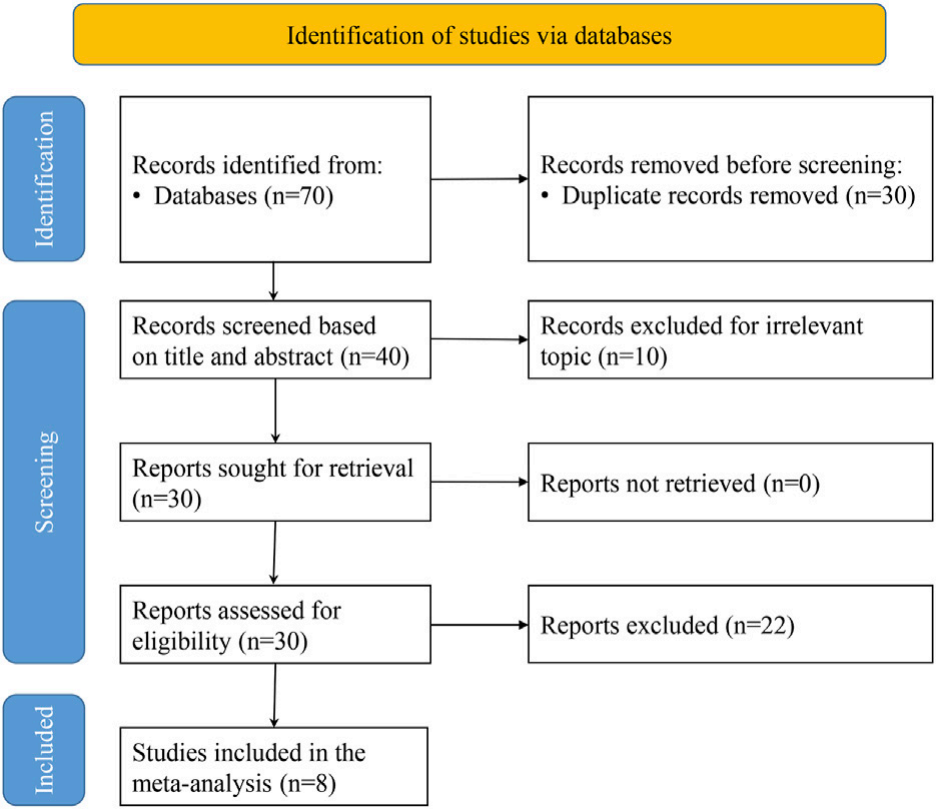
Flow chart of literature search.

Initially, 70 relevant articles were selected through a comprehensive review of 6 electronic databases. Ultimately, eight papers were considered for inclusion in our analysis (Jiang et al., 2022; Li et al., 2024a; Zhang et al., 2023; Du et al., 2020; Li et al., 2024b; Lin et al., 2022; Su, 2024; Tusheng et al., 2024) (Figure 1).

### Study characteristics

All eligible studies were observational and conducted in China. A total of 717 participants received LDH between 2020 and 2024, including 349 cases in the treatment group and 368 cases in the control group (Table 1).

**TABLE 1:** Study characteristics.

Author	Publication year	Country	Study design	Sample size		Age (years)		Follow-up (months)	
				Treatment	Control	Treatment	Control	Treatment	Control
Du	2020	China	OS	20	20	47.80 ± 13.46	45.85 ± 10.45	24.2 ± 1.9	24.2 ± 1.9
Jiang	2022	China	OS	51	57	48.1 ± 10.25	45.9 ± 9.83	12	12
Lin	2022	China	OS	36	37	48.36 ± 9.53	49.12 ± 8.72	15.37 ± 1.92	15.37 ± 1.92
Li	2023	China	OS	38	47	43.84 ± 13.15	41.79 ± 12.05	28.16 ± 2.59	27.96 ± 2.40
Zhang	2023	China	OS	50	48	41.5 ± 9.8	45.8 ± 11.0	18	18
Li	2024	China	OS	75	80	43.61 ± 11.72	44.25 ± 11.56	12	12
Li	2024	China	OS	30	30	37.1 ± 11.0	36.9 ± 10.7	27.2 ± 2.0	27.2 ± 2.0
Su	2024	China	OS	49	49	46.18 ± 8.92	46.85 ± 9.28	12	12

OS, observational study.

### Quality assessment

Information on the quality evaluation was shown in Table 2. Each article scored at least 7 and was categorized as high quality.

**TABLE 2:** Newcastle-Ottawa Scale of included observational studies.

Study	Selection	Comparability	Outcome	Total score
Du 2020	3	1	3	7
Jiang 2022	4	1	3	8
Lin 2022	4	1	3	8
Li 2023	4	1	3	8
Zhang 2023	4	1	3	8
Li 2024A	4	1	3	8
Li 2024B	4	1	3	8
Su 2024	3	1	3	7

### Meta-analysis results

#### VAS

**FIGURE 2:**
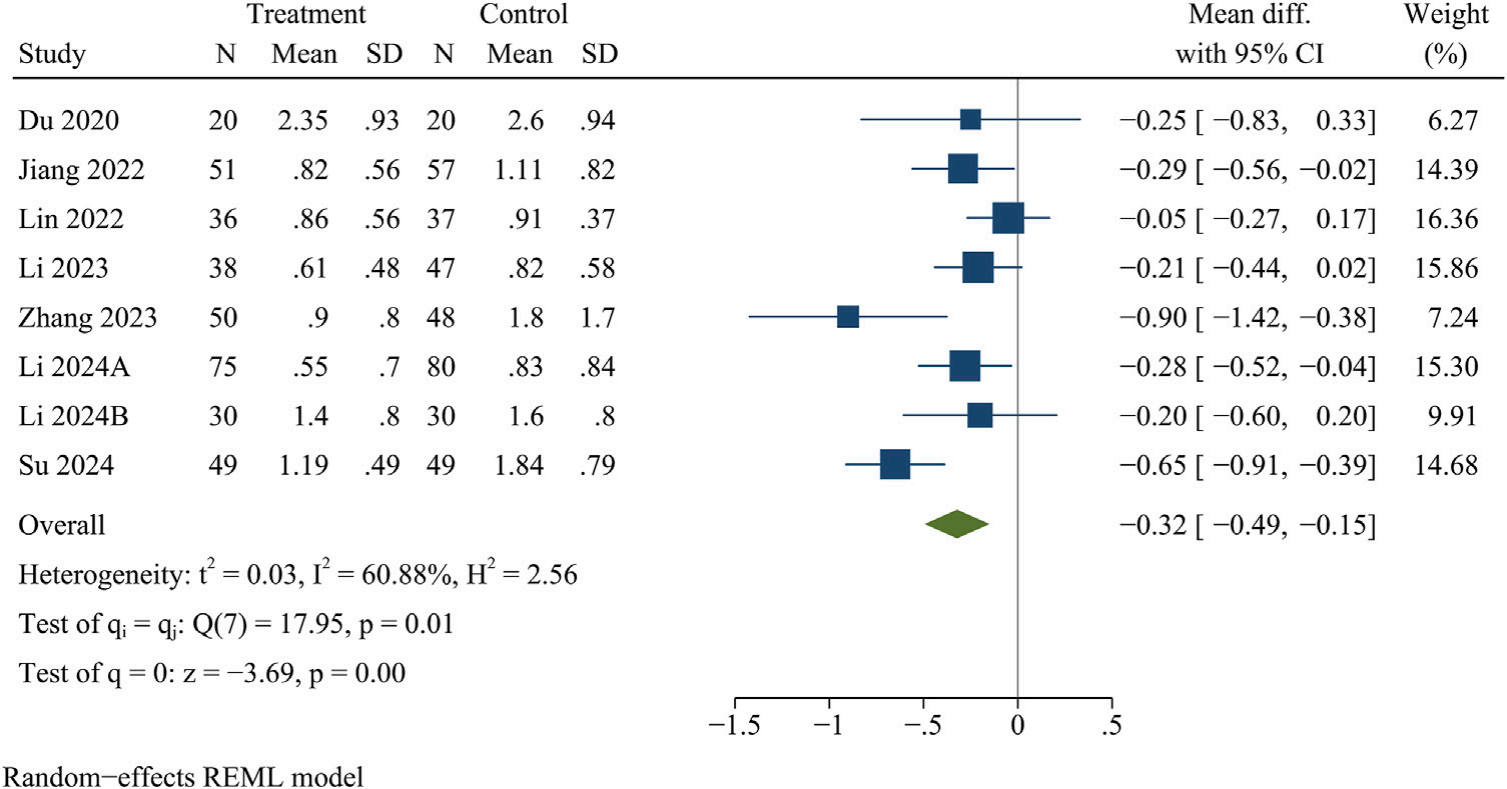
Forest plot of visual analogue score for back pain.

**FIGURE 3:**
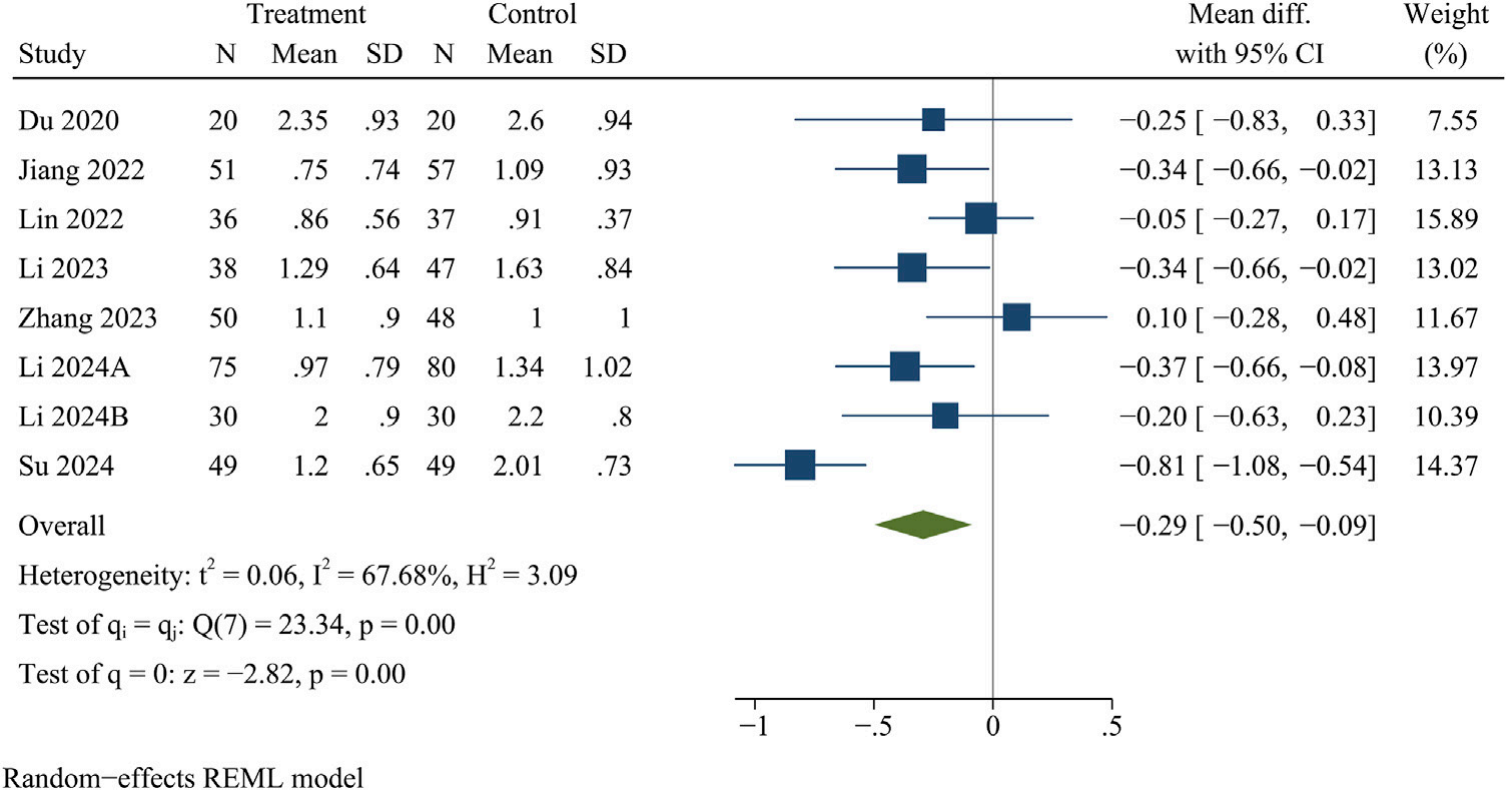
Forest plot of visual analogue score for leg pain.

Eight studies reported VAS for back pain and leg pain separately, including 717 patients (349 in the treatment group and 368 in the control group). Ultimately, the results of the meta-analysis showed that at the final follow-up, the treatment group had significantly less VAS for back pain [WMD = −0.32, 95% CI (−0.49, −0.15), *p* < 0.01] (Figure 2) and VAS for leg pain [WMD = −0.29, 95% CI (−0.50, −0.09), *p* < 0.01] (Figure 3) than the control group (Table 3).

**TABLE 3:** Findings of meta-analysis.

Outcomes	Study	Effect size	95% CI		P-value	Heterogeneity		Effects	Egger test
	Size	WMD/OR	Lower limit	Upper limit		I^2^ (%)	*P*-value	Model	*P*-value
VAS for back pain	8	−0.32	−0.49	−0.15	<0.01	60.88	0.01	Random	0.28
VAS for leg pain	8	−0.29	−0.50	−0.09	<0.01	67.68	<0.01	Random	0.67
JOA scores	5	1.82	0.09	3.55	0.04	93.20	<0.01	Random	0.51
ODI	8	−2.46	−4.56	−0.36	0.02	90.10	<0.01	Random	0.82
IDH	5	0.73	0.50	0.95	<0.01	1.01	0.40	Fixed	0.19
IDP	2	−0.13	−0.23	−0.04	<0.01	47.47	0.17	Fixed	0.17
SCSA	2	9.85	5.04	14.66	<0.01	46.28	0.17	Fixed	0.17
RVG	3	1.69	0.82	2.56	<0.01	0.00	0.37	Fixed	0.16
Recurrence	4	0.27	0.10	0.70	0.01	0.00	0.92	Fixed	0.91

VAS, visual analogue score; IDH, intervertebral disc height; IDP, intervertebral disc protrusion; ODI, oswestry disability index; JOA, japanese orthopaedic association; SCSA, spinal canal cross-sectional area; RVG, ratio value of disc grey scales; WMD, weighted mean difference; CI, confidence intervals; OR, odds ratio.

#### JOA scores

**FIGURE 4:**
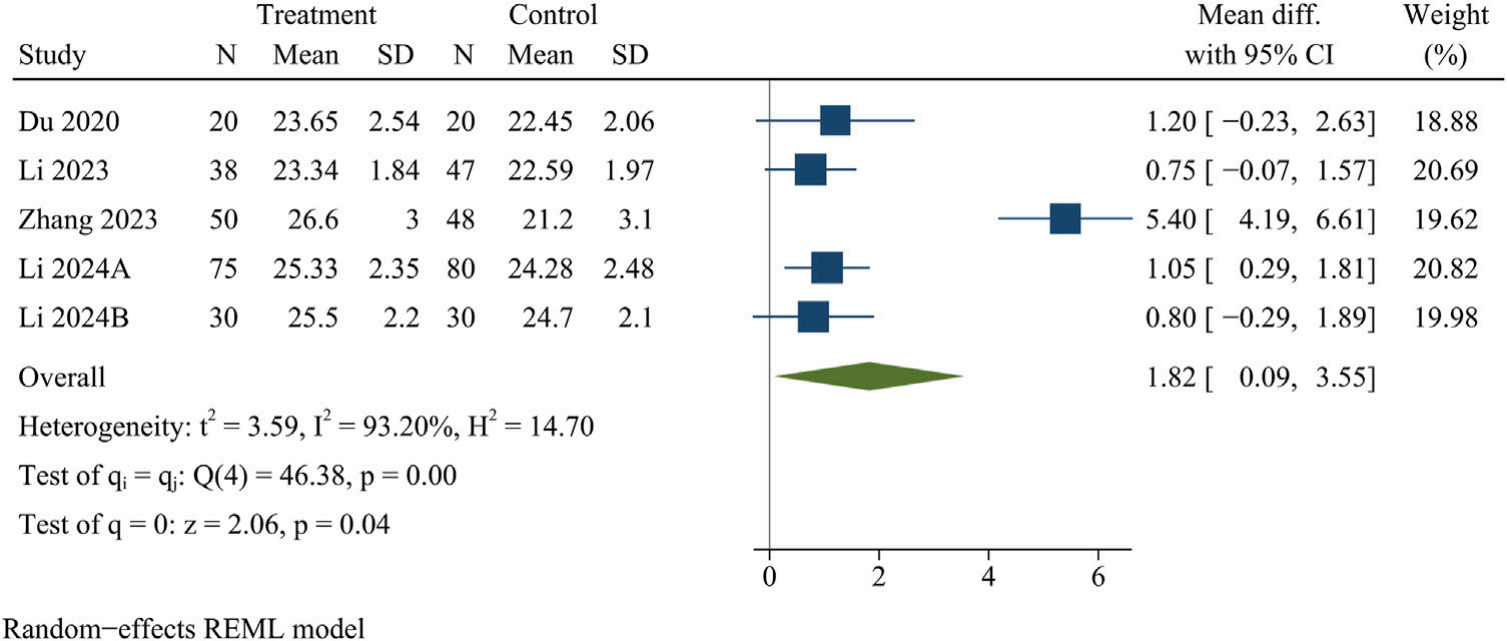
Forest plot of Japanese Orthopaedic Association scores.

JOA scores were recorded in five papers, including 438 patients. At the final follow-up, the treatment group provided higher JOA scores than the control group [WMD = 1.82, 95% CI (0.09, 3.55), *p* = 0.04] (Figure 4).

#### ODI

**FIGURE 5:**
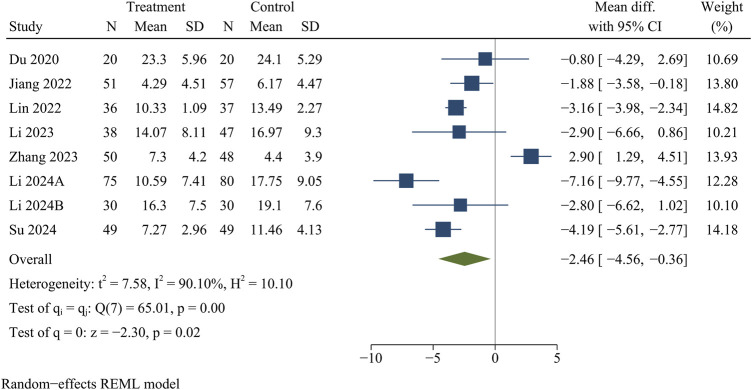
Forest plot of Oswestry disability index.

Eight studies analyzed ODI data. At the final follow-up, the treatment group had a significantly smaller ODI than the control group [WMD = −2.46, 95% CI (−4.56, −0.36), *p* = 0.02] (Figure 5).

#### IDH

**FIGURE 6:**
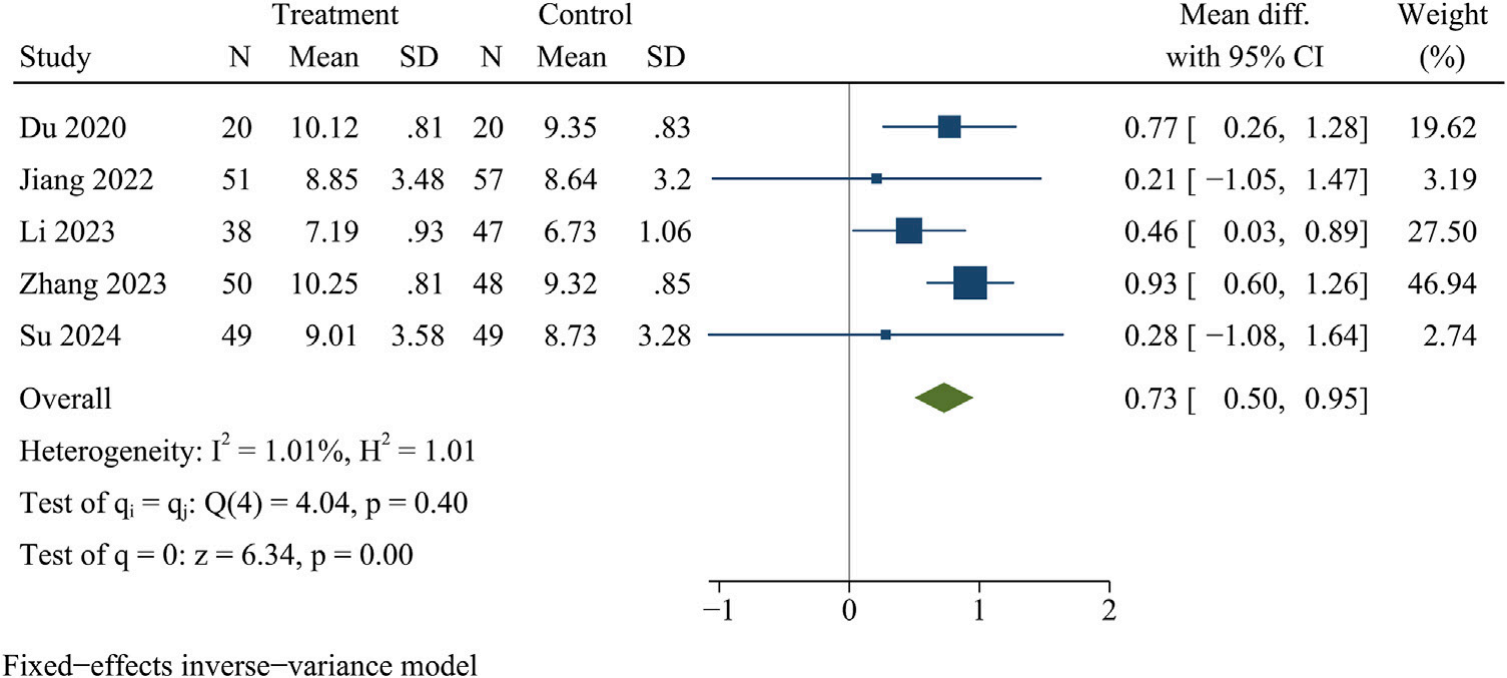
Forest plot of intervertebral disc height.

Five publications recorded information on IDH, including 208 in the treatment group and 221 in the control group. At the final follow-up, IDH was significantly greater in the treatment group than in the control group [WMD = 0.73, 95% CI (0.50, 0.95), *p* < 0.01] (Figure 6).

#### IDP

**FIGURE 7:**
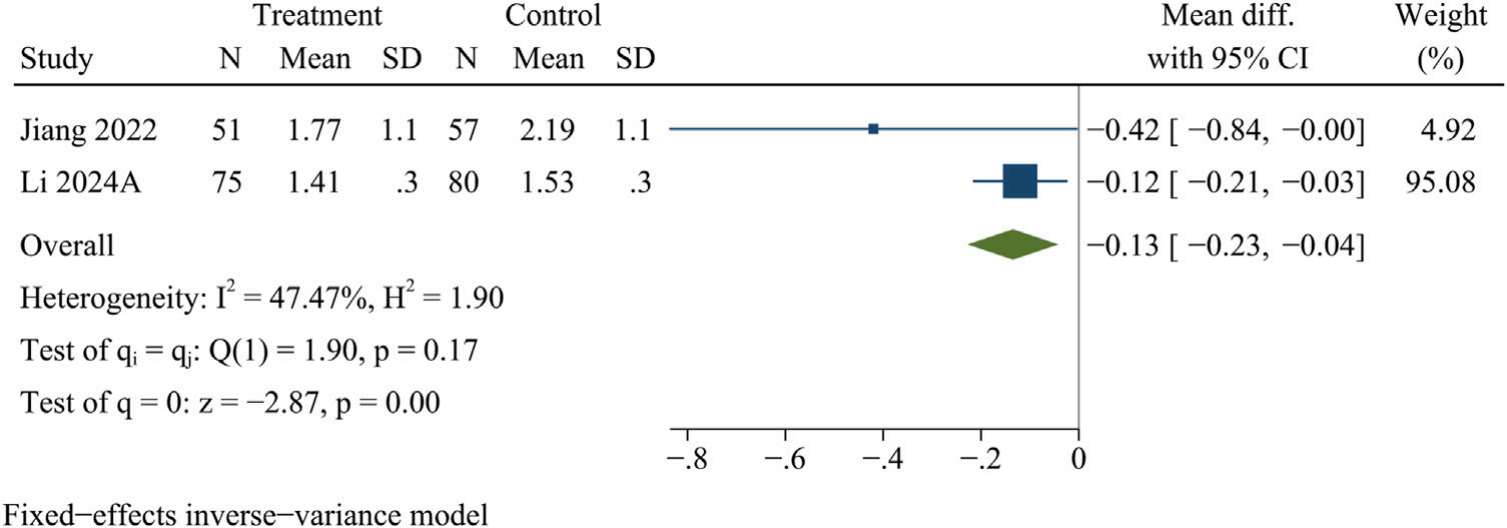
Forest plot of intervertebral disc protrusion.

A total of 263 participants in 2 studies were evaluated for IDP data. At the final follow-up, the control group showed more pronounced disc protrusion than the treatment group [WMD = −0.13, 95% CI (−0.23, −0.04), *p* < 0.01] (Figure 7).

#### SCSA

**FIGURE 8:**
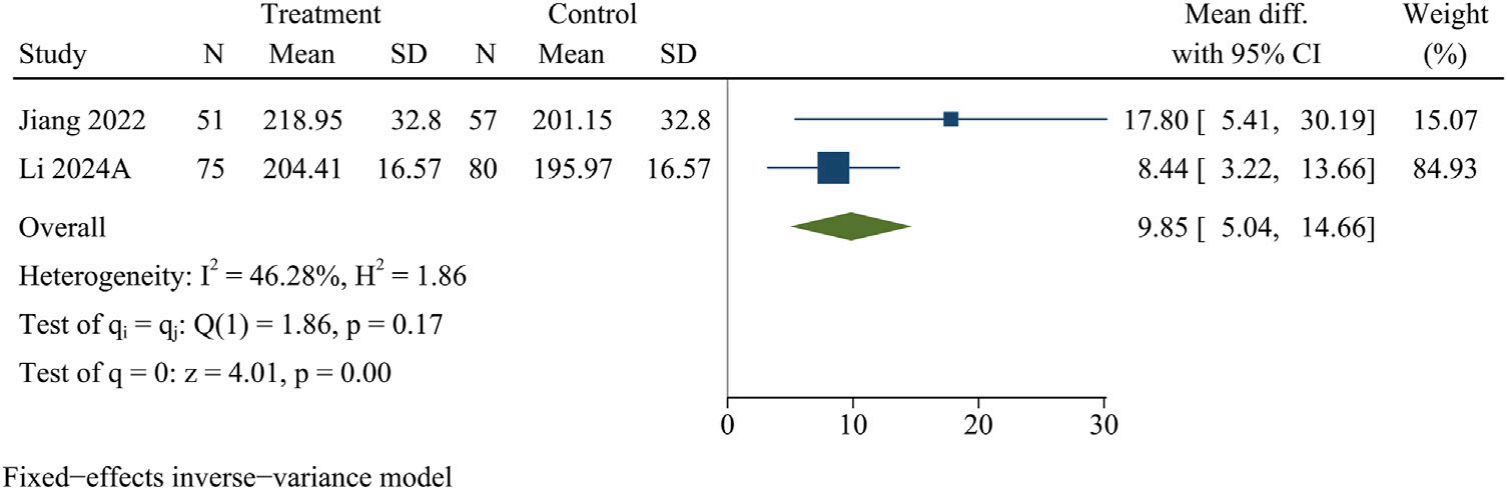
Forest plot of spinal canal cross-sectional area.

Two research studies contained information on SCSA, consisting of 126 in the treatment group and 137 in the control group. At the final follow-up, the treatment group obtained more SCSA than the control group [WMD = 9.85, 95% CI (5.04, 14.66), *p* < 0.01] (Figure 8).

#### RVG

**FIGURE 9:**
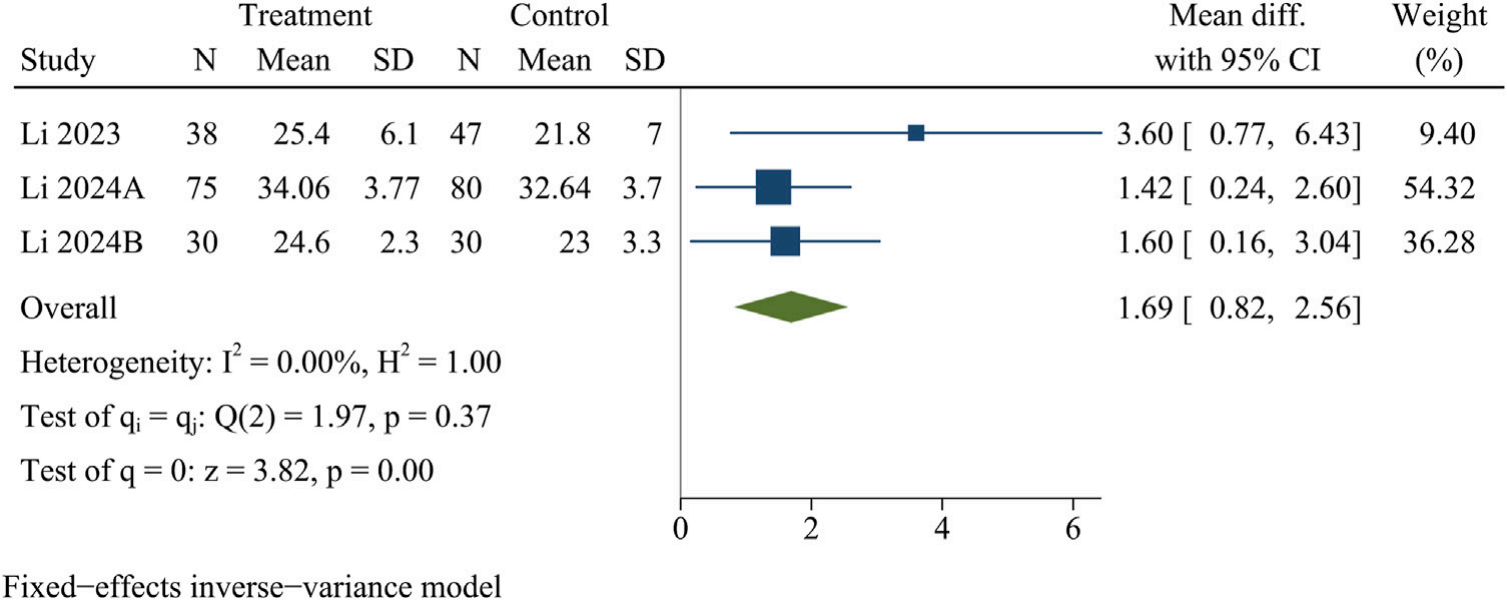
Forest plot of ratio value of disc grey scales.

Three studies recorded RVG. At the last follow-up, the treatment group had a significantly higher RVG than the control group [WMD = 1.69, 95% CI (0.82, 2.56), *p* < 0.01] (Figure 9).

### Recurrence

**FIGURE 10:**
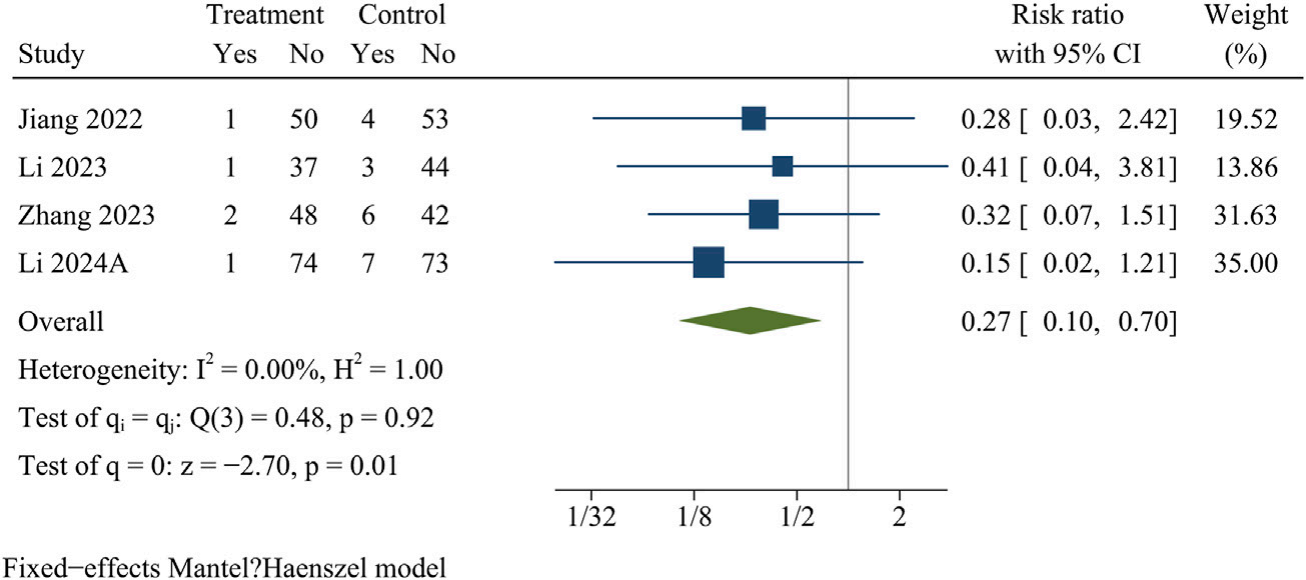
Forest plot of recurrence rate.

Recurrence information was recorded in four papers comprising 446 patients (treatment group 214, control group 232). At the final follow-up, the recurrence rate was significantly lower in the treatment group than in the control group [OR = 0.27, 95% CI (0.10, 0.70), *p* = 0.01] (Figure 10).

### Sensitivity analyses

Sensitivity analyses showed that the results of meta-analyses were robust for all outcome indicators except JOA scores and ODI (Supplementary File 2).

### Publication bias

Egger test was used to detect publication bias, and the findings demonstrated no publication bias within our study (*p* > 0.05) (Supplementary File 3).

## Discussion

In recent years, the incidence of LDH increased with age, which seriously affected people’s health and quality of life. Surgical intervention was a necessary treatment for patients with LDH who could not achieve satisfactory results with conservative treatment. Conventional open operation was the classical therapy in treating LDH, however, it required extensive resection of the vertebral laminas and facet joints, as well as extensive stripping of the paraspinal muscle, which could easily induce complications such as lumbar postoperative failure syndrome. With the popularity of minimally invasive concept, minimally invasive spinal endoscopy became one of the mainstream surgical procedures for the treatment of LDH. PELD was widespread applied for treating LDH, and could achieve satisfactory clinical efficacy. However, minimally invasive spinal endoscopy could not essentially delay disc degeneration and could lead to further aggravation of disc degeneration after surgery. In addition, the non-vascular nature of the intervertebral disc made the repair ability after disc degeneration poor. Therefore, how to slow down disc degeneration and enhance disc repair and regeneration was a key clinical problem that needed to be solved urgently.

With many scholars exploring the mechanism of intervertebral disc degeneration, PRP was gaining more and more attention as a new, green regenerative therapy. PRP was a platelet concentrate obtained from fresh whole blood after centrifugal separation, and platelets could release a variety of growth factors after activation, such as platelet-derived growth factor, vascular endothelial growth factor, insulin-like growth factor, transforming growth factor β, epidermal growth factor and so on, which had been confirmed to have the effects of promoting the remodeling and regeneration of intervertebral discs, angiogenesis, anti-inflammation and so on (Luan et al., 2025). At present, PRP had been extensively utilized for treating lumbar diseases, achieving favorable clinical efficacy (Zhang et al., 2024).

To date, there were few studies about PRP injections combined with PELD for the treatment of LDH. Nevertheless, some papers reported clinical findings after intradiscal PRP injection for treating lumbar diseases (Zhang et al., 2024; Kaux et al., 2024). A meta-analysis found intradiscal injections led to statistically significant improvements in the VAS, with lower incidence of complications and reinjections (Hirase et al., 2020). To the best of our knowledge, our study was the first meta-analysis to comprehensively compare the effectiveness of PELD combined with PRP injections for LDH. In this study, we found PELD combined with PRP could provide lower VAS, ODI, IDP, and recurrence rates, and higher JOA scores, IDH, SCSA, and DVG, compared with PELD alone. Our findings suggested that intraoperative PRP injection following PELD might benefit annulus fibrosus repair, delay intervertebral disc degeneration and improve clinical outcomes.

The main principle of PRP in repairing degenerated discs was to inject a high concentration of platelets directly into the disc to initiate a healing cascade. The fibronectin in PRP provided a scaffolding structure for cell proliferation and tissue repair, preventing cell loss while adhering to the annulus fibrosus cells to further seal the annulus fibrosus fracture. Previous animal experiments demonstrated PRP could significantly suppress inflammation mediated by inflammatory mediators and pro-degradative enzymes, thus hindering progressive intervertebral disc degeneration (Tao et al., 2024; Luan et al., 2025).

In 2011, Akeda et al. were the first to demonstrate autologous PRP applied within the intervertebral disc was a secure and beneficial biotherapy in treating lumbar degenerative diseases. Since then, PRP had been extensively employed for treating lumbar disc disease and achieved encouraging clinical results. In this study, VAS, ODI and JOA scores of LDH patients treated with PRP improved more significantly, indicating that PRP could help to relieve pain and improve the quality of life. The efficacy of PRP injections seemed to be associated not only with the interaction of local inflammatory factors, but also with the repairs of local damage. Those receiving PRP injections exhibited better performance in pain management, while PRP could reduce postsurgical pain and numbness. We suggested that injecting PRP around a lesion might release numerous factors, which might alleviate inflammation as well as relieve symptom. In our study, there were significant differences regarding ODI between both groups, which could be related to multiple factors released from PRP, playing a role in interference with scar formation surrounding neural tissue. The impact of PRP infiltration on extracellular matrix synthesis, anti-inflammatory mechanisms, analgesia and subchondral bone homeostasis might facilitate intervertebral disc repair. In summary, these might boost endogenous repair mechanisms and enhance recovery.

Discectomy unavoidably led to subsequent disc degeneration. IDH loss was a natural process of intervertebral disc degeneration following discectomy. A previous investigation showed PRP injections prevented the reduction of DH in a rabbit model (Gui et al., 2015). The results of this previous trial were comparable to our findings. At the last follow-up, LDH patients with PRP injection showed a smaller decrease in IDH, suggesting that PRP was able to delay disc degeneration to some extent.

Residual annulus noted by MRI was prevalent in the early postoperative period following PELD. Variations in DVG and IDP over time suggested the possibility of disc remodeling (Mahatthanatrakul et al., 2019). In our study, the PRP group had less disc protrusion and greater SCSA and DVG during the follow-up period, suggesting that PRP played an active role in annulus fibrosus remodeling. These changes led to enlargement in spinal canal space and reduction of nerve root compression and irritation, which explained the alleviation of clinical symptom in a different way.

In our study, the incidence of recurrence was significantly less in the treatment group, suggesting PRP could contribute to the prevention of recurrence. Ideal annulus fibrosus remodeling could effectively establish a mechanical barrier to protect against subsequent recurrence of disc protrusion, decrease the appearance of recompression, as well as extending the spinal canal, avoiding the formation of postoperative fibrotic scarring in the spinal canal, and minimizing the release of inflammation factors, thus achieving favorable effects and preventing the incidence of postsurgical pains and numbness. Following proper repair of the annulus fibrosus, the intervertebral disc could be regarded as a sealed area, which prevented the leakage of biological agents and provided the foundation for biologic therapy of the intervertebral disc. Also, homeostasis of discs was the rationale for avoidance of recurrence (Li et al., 2021).

Meanwhile, the safety of PRP was also a major concern. Because PRP was derived from autologous blood without immune rejection, it had limited potential for infection and allergic reaction (Xie et al., 2020). It was also reported that PRP had anti-microbial properties, which helped to reduce the risk of infection after surgery (Mohammed and Yu, 2018). Du et al. reported one patient in the observation group suffered from aggravation of radicular symptom on the next day after treatment, which was considered to be caused by increased disc pressure after the placement of PRP gel microspheres or irritation of the nerve root by a small amount of inflammatory factors such as leukocytes in the PRP, and the symptoms were relieved after symptomatic treatment, and there was no neurological damage such as decreased sensory muscle power in the lower extremities (Du et al., 2020).

There were still different opinions on the form of PRP to be used. Bhatia et al. injected 5 mL of PRP solution into nerve root region around diseased segments by the epidural route, and the follow-up results showed a significant improvement in the patients’ VAS scores and straight leg raising test within 3 months (Bhatia and Chopra, 2016). In order to prevent spillage of PRP solution, Vadalà et al. designed a new injectable hydrogel composed of PRP, hyaluronic acid and patulinase, and biological tests showed that this preparation improved the cell viability and proliferation of their MSCs (Vadala et al., 2017). Li et al. reported that PRP injection into the gel capsule enabled the slow release of high concentrations of growth factors in PRP and their sustained action on intervertebral disc cells, which greatly improved the therapeutic effect of PRP in repairing degenerated intervertebral discs (Li et al., 2024b).

## Limitations

Our study had several limitations. Firstly, the limited sample size might impact the representativeness of the study results. Secondly, variations in PRP preparation techniques, as well as differences in injection dosage and composition, could introduce bias into the results. Thirdly, all eligible studies were conducted in China, which might impose geographic and healthcare system constraints on generalizability. Fourthly, incomplete reporting of complications in some studies might have led to an underestimation of risk. Finally, the follow-up period was insufficient to comprehensively assess the long-term benefits and risks associated with PRP application. Therefore, future research should aim to extend follow-up duration and increase sample sizes to further validate the long-term efficacy and safety of PRP therapy. Additionally, it is important to note that active components and concentrations of PRP extracted through different methods can vary significantly. Future investigations should focus on determining optimal PRP concentration and injection dosage for promoting intervertebral disc repair, as well as identifying implantation methods and treatment durations that maximize the therapeutic potential of PRP.

## Conclusion

Our study demonstrated that compared with PELD alone, PELD combined with PRP significantly reduced VAS scores, ODI, IDP and recurrence rates while increasing JOA scores, IDH, SCSA and DVG. In conclusion, these findings indicate that for the treatment of LDH, PELD combined with PRP injection markedly enhances clinical efficacy while reducing recurrence rates compared to PELD alone. Moving forward, larger multicenter randomized controlled trials with extended follow-up periods are necessary to confirm the long-term efficacy and safety of this combination therapy.

## Abbreviations

LDH, lumbar disc herniation; PRP, platelet-rich plasma; PELD, percutaneous endoscopic lumbar discectomy; VAS, visual analogue score; IDH, intervertebral disc height; IDP, intervertebral disc protrusion; ODI, Oswestry disability index; JOA, Japanese Orthopaedic Association; SCSA, spinal canal cross-sectional area; RVG, ratio value of disc grey scales; WMD, weighted mean difference; CI, confidence intervals; OR, odds ratio.
