# Supplementary material for: Efficacy of platelet-rich plasma injection with percutaneous endoscopic lumbar discectomy for lumbar disc herniation: a systematic review and meta-analysis
Source: Front Pharmacol. 2025 Sep 3;16:1622974. doi: 10.3389/fphar.2025.1622974 (PMC12441161; doi:10.3389/fphar.2025.1622974)
Supplement: Supplementary file 1 [file DataSheet2.pdf]

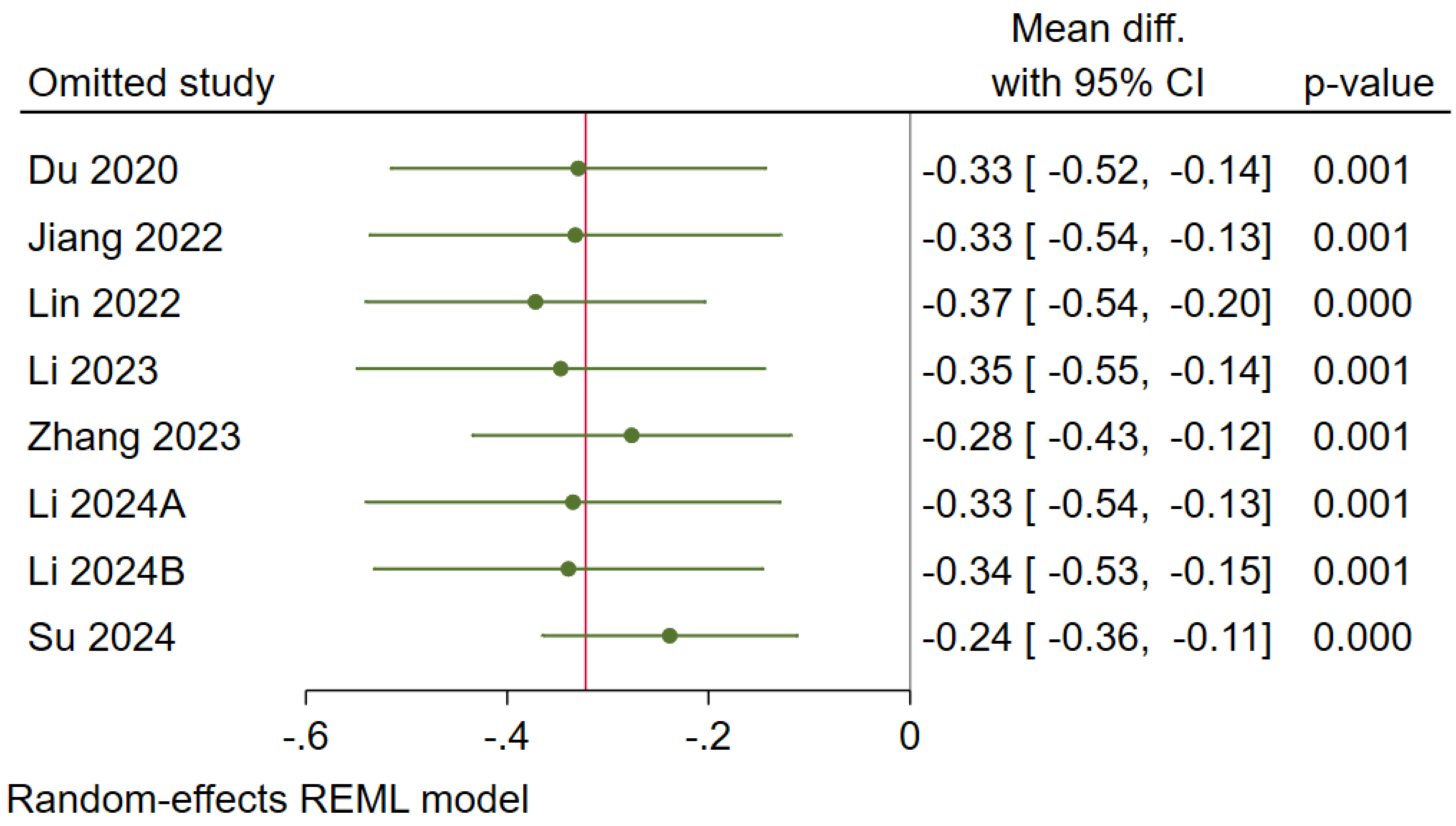

Sensitivity analysis of visual analogue score for back pain

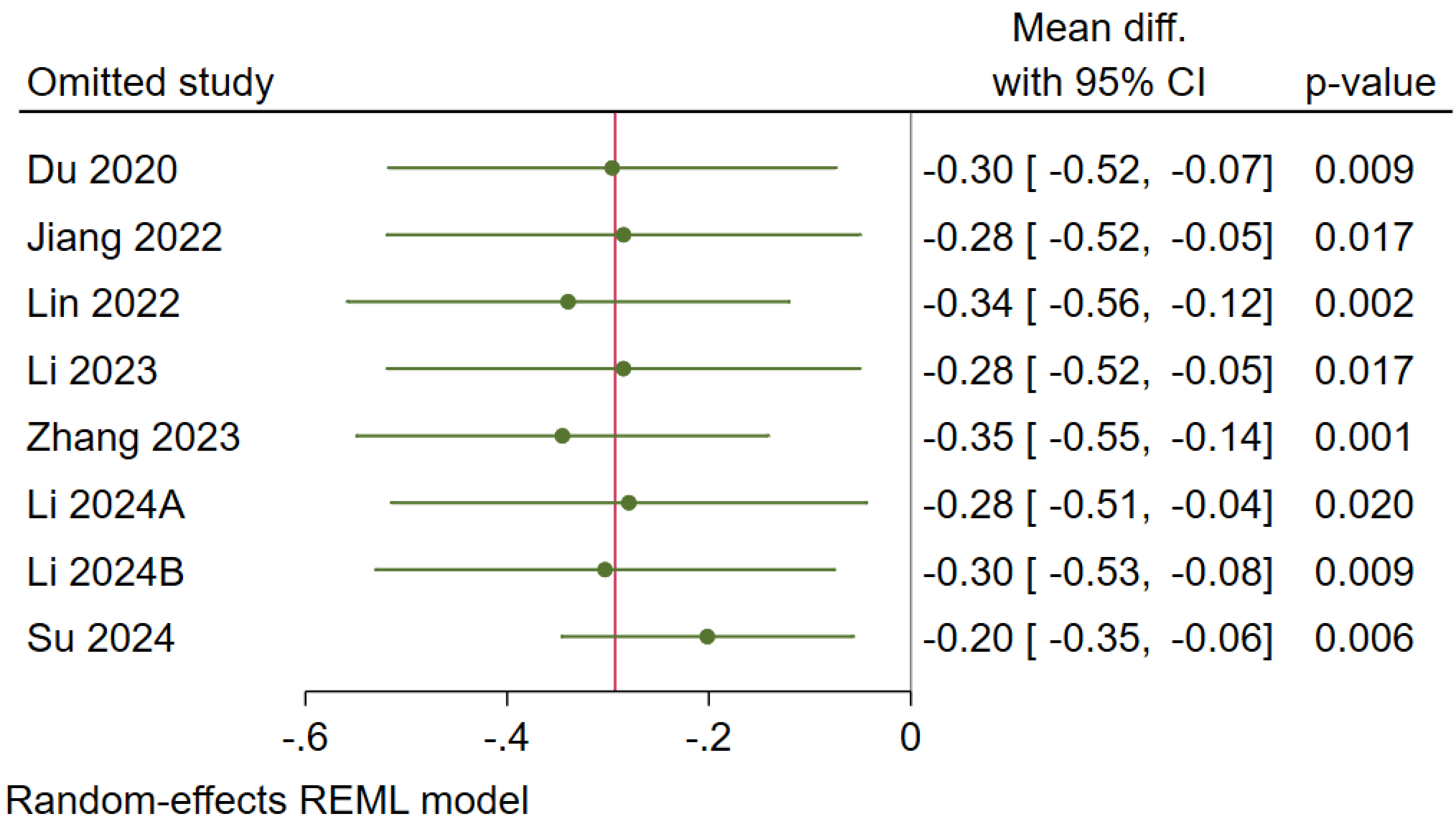

Sensitivity analysis of visual analogue score for leg pain

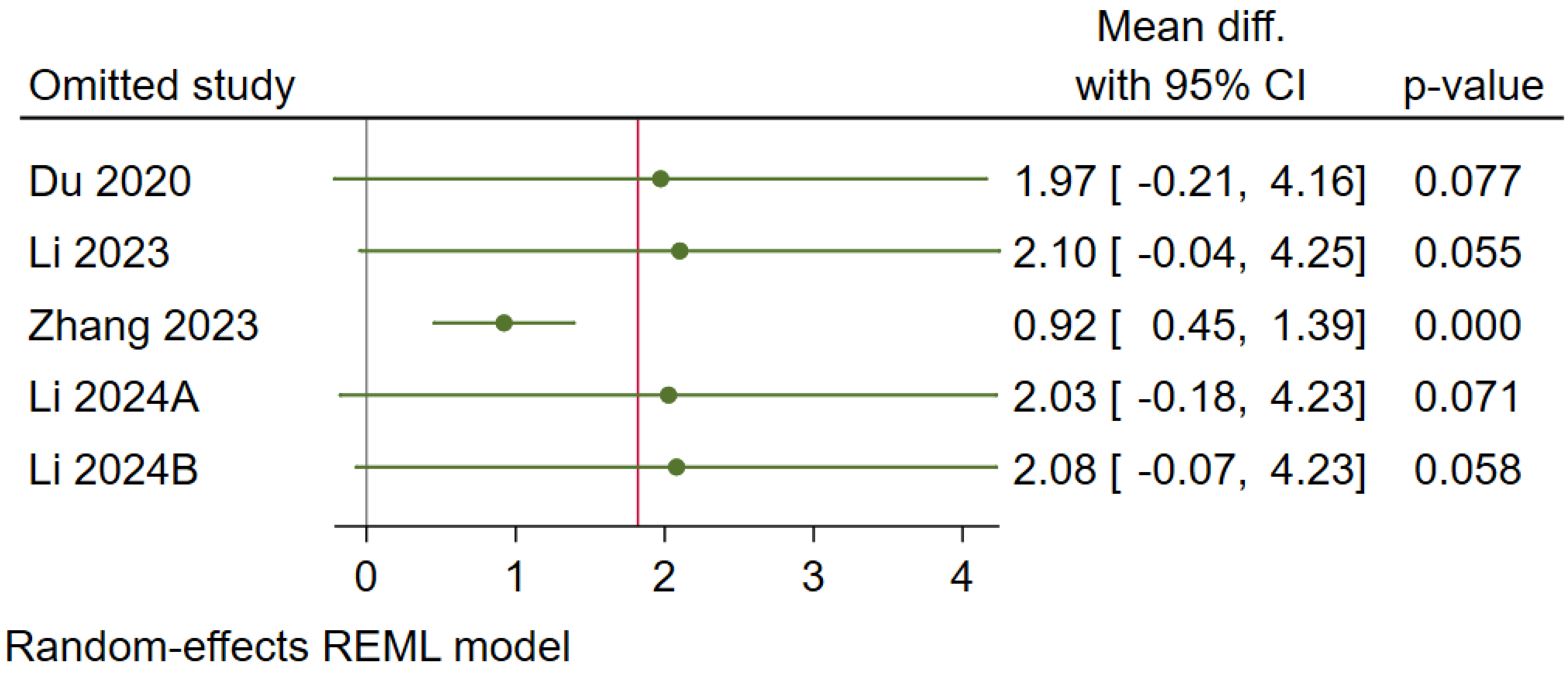

Sensitivity analysis of Japanese Orthopaedic Association scores

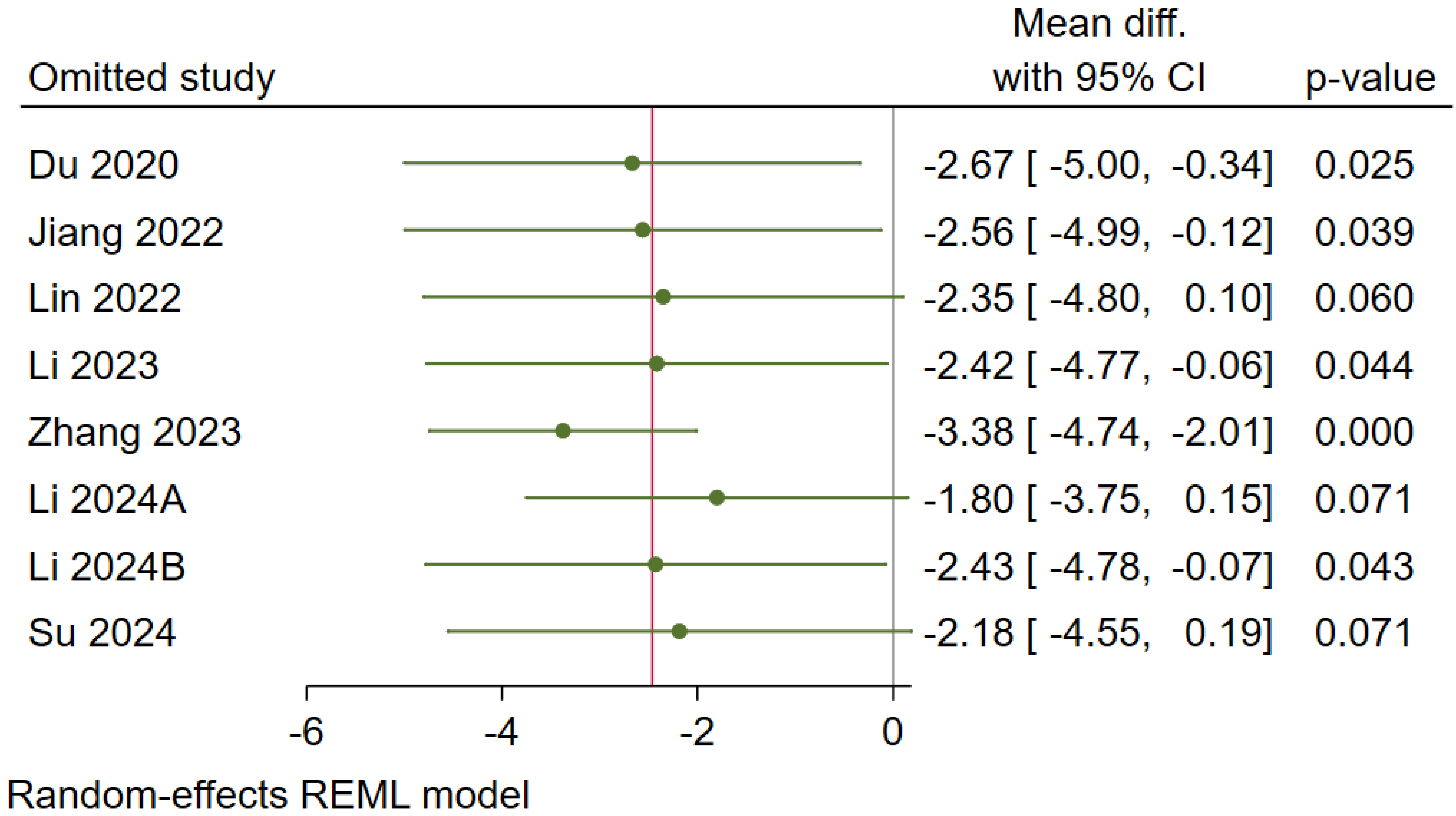

Sensitivity analysis of Oswestry disability index

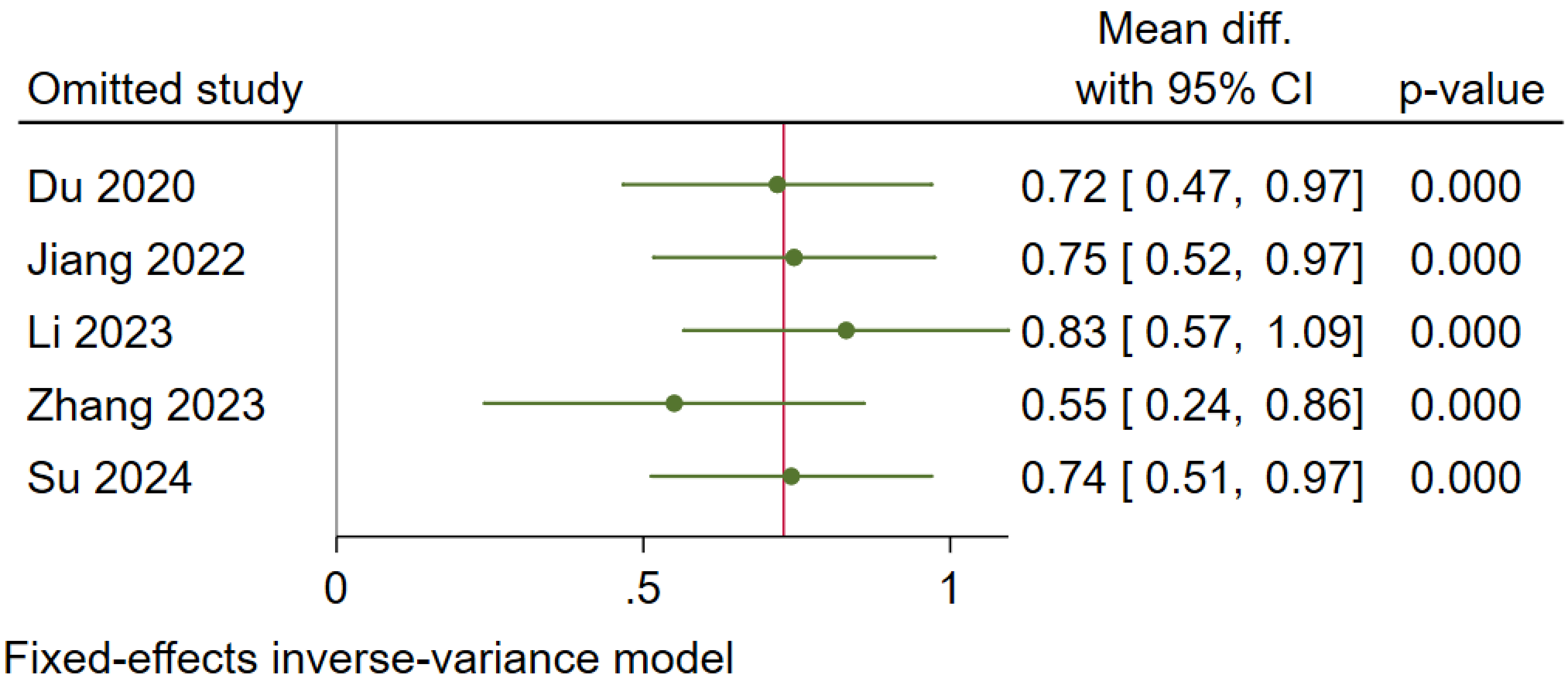

Sensitivity analysis of intervertebral disc height

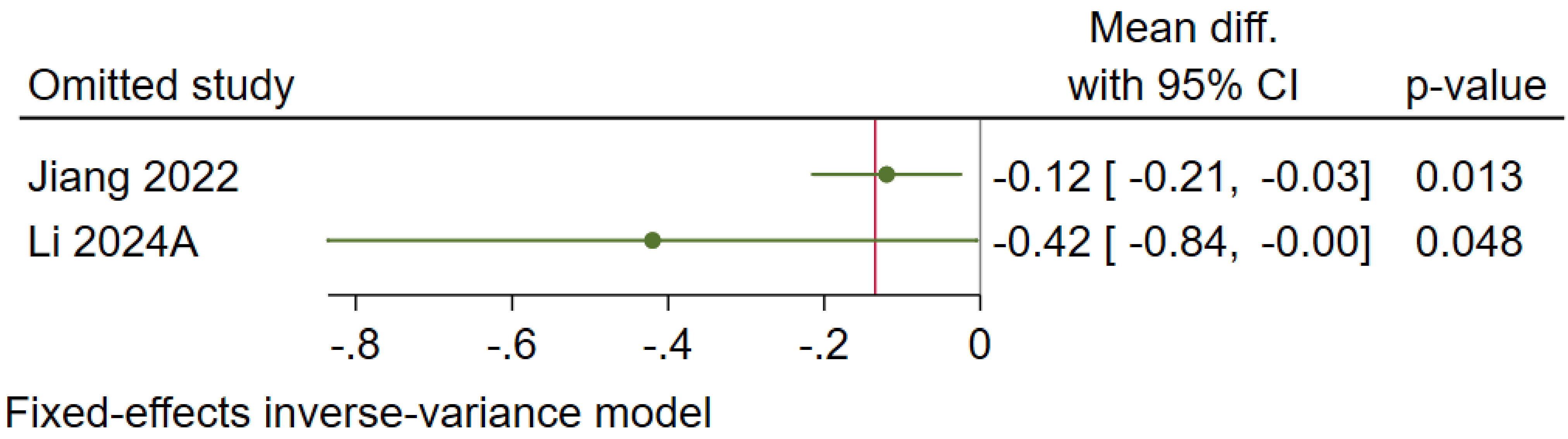

Sensitivity analysis of intervertebral disc protrusion

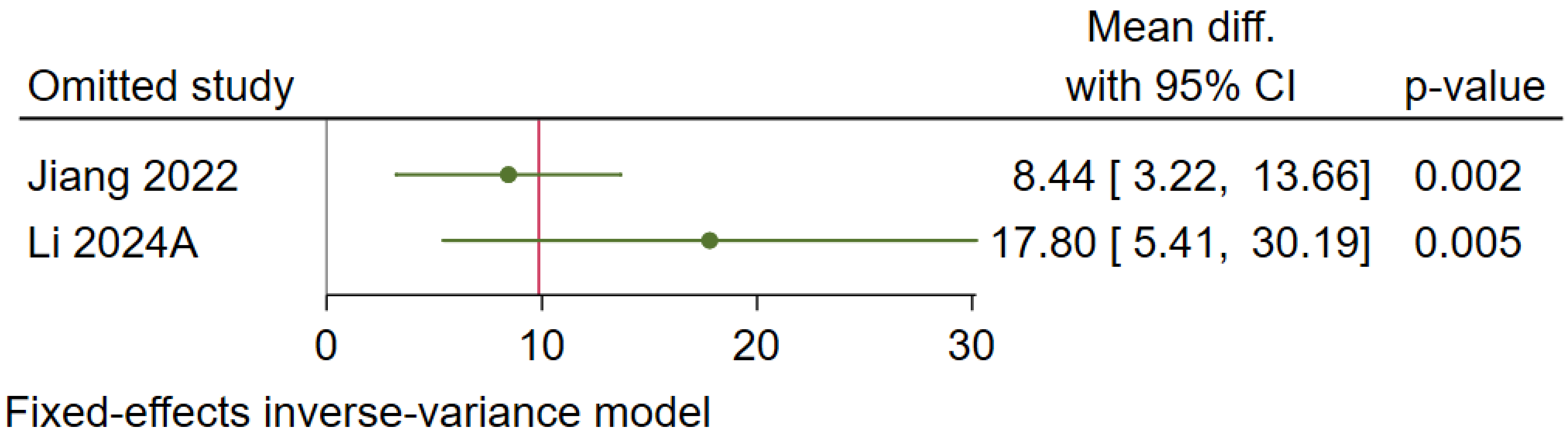

Sensitivity analysis of spinal canal cross-sectional area

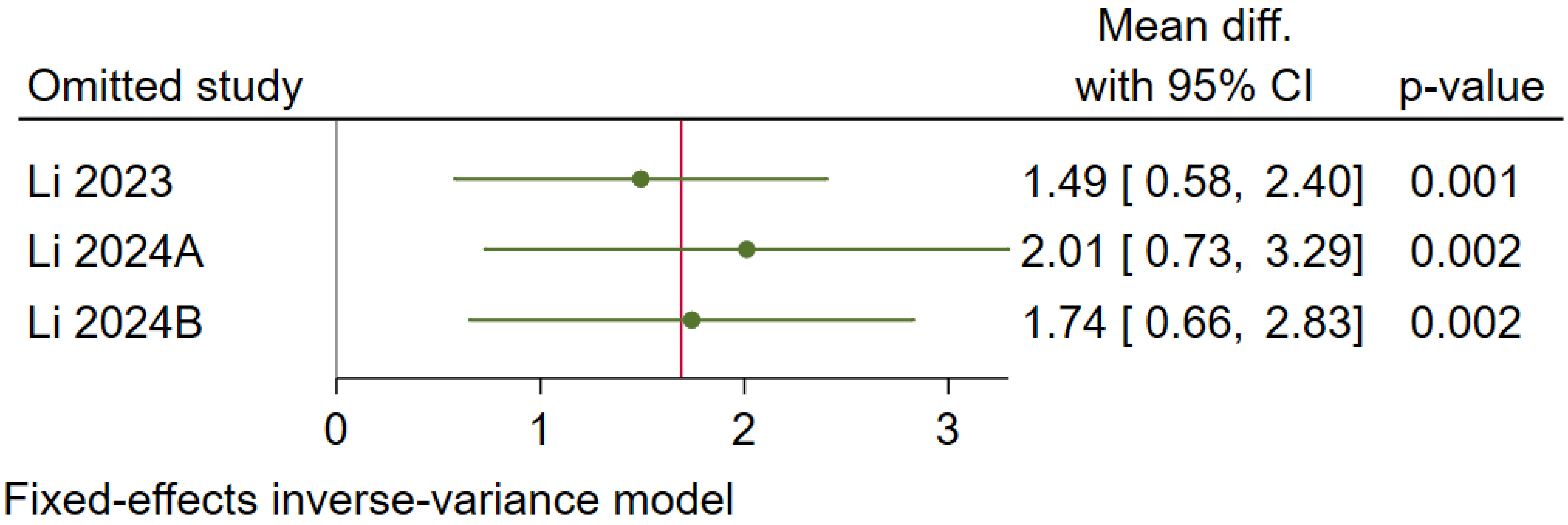

Sensitivity analysis of ratio value of disc grey scales

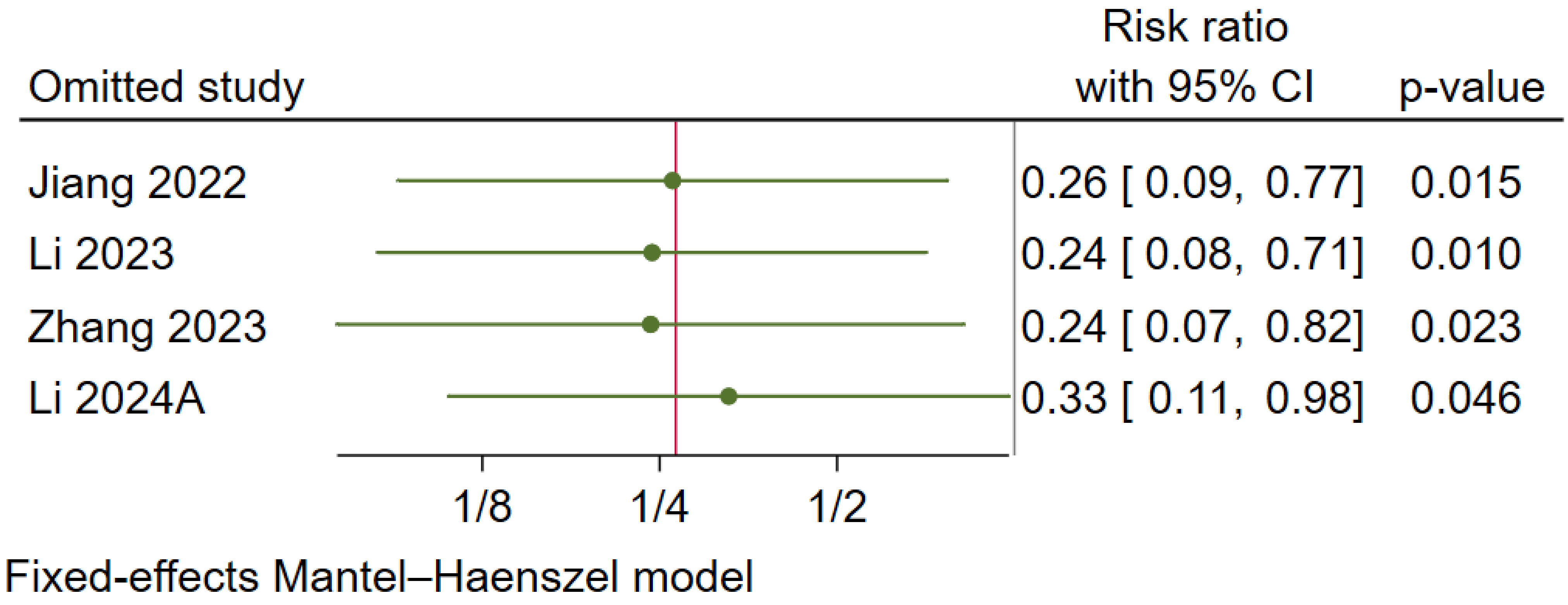

Sensitivity analysis of recurrence rate
