# Supplementary material for: Efficacy of platelet-rich plasma injection with percutaneous endoscopic lumbar discectomy for lumbar disc herniation: a systematic review and meta-analysis
Source: Front Pharmacol. 2025 Sep 3;16:1622974. doi: 10.3389/fphar.2025.1622974 (PMC12441161; doi:10.3389/fphar.2025.1622974)
Supplement: Supplementary file 2 [file DataSheet3.pdf]

```
. meta bias, egger

  Effect-size label: Mean diff.
        Effect size: _meta_es
          Std. err.: _meta_se

Regression-based Egger test for small-study effects
Random-effects model
Method: REML

H0: beta1 = 0; no small-study effects
            beta1 =     -1.65
      SE of beta1 =     1.512
                z =     -1.09
       Prob > |z| =    0.2753

.
```

Publication bias of visual analogue score for back pain

```
. meta bias, egger

  Effect-size label: Mean diff.
        Effect size: _meta_es
          Std. err.: _meta_se

Regression-based Egger test for small-study effects
Random-effects model
Method: REML

H0: beta1 = 0; no small-study effects
            beta1 =      0.97
      SE of beta1 =     2.265
                z =      0.43
       Prob > |z| =    0.6691

.
```

Publication bias of visual analogue score for leg pain

```
. meta bias, egger

  Effect-size label: Mean diff.
        Effect size: _meta_es
          Std. err.: _meta_se

Regression-based Egger test for small-study effects
Random-effects model
Method: REML

H0: beta1 = 0; no small-study effects
            beta1 =      4.95
      SE of beta1 =     7.516
                z =      0.66
       Prob > |z| =    0.5101

.
```

Publication bias of Japanese Orthopaedic Association scores

```
. meta bias, egger

  Effect-size label: Mean diff.
        Effect size: _meta_es
          Std. err.: _meta_se

Regression-based Egger test for small-study effects
Random-effects model
Method: REML

H0: beta1 = 0; no small-study effects
            beta1 =     -0.48
      SE of beta1 =     2.073
                z =     -0.23
       Prob > |z| =    0.8182

.
```

Publication bias of Oswestry disability index

```
. meta bias, egger

  Effect-size label: Mean diff.
        Effect size: _meta_es
          Std. err.: _meta_se

Regression-based Egger test for small-study effects
Fixed-effects model
Method: Inverse-variance

H0: beta1 = 0; no small-study effects
            beta1 =     -1.30
      SE of beta1 =     0.994
                z =     -1.31
       Prob > |z| =    0.1905

.
```

Publication bias of intervertebral disc height

```
. meta bias, egger

  Effect-size label: Mean diff.
        Effect size: _meta_es
          Std. err.: _meta_se

Regression-based Egger test for small-study effects
Fixed-effects model
Method: Inverse-variance

H0: beta1 = 0; no small-study effects
            beta1 =     -1.83
      SE of beta1 =     1.327
                z =     -1.38
       Prob > |z| =    0.1677

.
```

Publication bias of intervertebral disc protrusion

```
. meta bias, egger

  Effect-size label: Mean diff.
        Effect size: _meta_es
          Std. err.: _meta_se

Regression-based Egger test for small-study effects
Fixed-effects model
Method: Inverse-variance

H0: beta1 = 0; no small-study effects
            beta1 =      2.56
      SE of beta1 =     1.875
                z =      1.36
       Prob > |z| =    0.1724

.
```

Publication bias of spinal canal cross-sectional area

```
. meta bias, egger

  Effect-size label: Mean diff.
        Effect size: _meta_es
          Std. err.: _meta_se

Regression-based Egger test for small-study effects
Fixed-effects model
Method: Inverse-variance

H0: beta1 = 0; no small-study effects
            beta1 =      2.58
      SE of beta1 =     1.854
                z =      1.39
       Prob > |z| =    0.1640

.
```

Publication bias of ratio value of disc grey scales

```
. meta bias, egger
note: declared Mantel–Haenszel method not supported with meta bias; using
      inverse-variance method

  Effect-size label: Log risk-ratio
        Effect size: _meta_es
          Std. err.: _meta_se

Regression-based Egger test for small-study effects
Fixed-effects model
Method: Inverse-variance

H0: beta1 = 0; no small-study effects
            beta1 =     -0.39
      SE of beta1 =     3.282
                z =     -0.12
       Prob > |z| =    0.9055

.
```

Publication bias of recurrence rate
