# Supplementary material for: Efficacy of platelet-rich plasma injection with percutaneous endoscopic lumbar discectomy for lumbar disc herniation: a systematic review and meta-analysis
Source: Front Pharmacol. 2025 Sep 3;16:1622974. doi: 10.3389/fphar.2025.1622974 (PMC12441161; doi:10.3389/fphar.2025.1622974)
Supplement: Supplementary file 3 [file DataSheet1.pdf]

The definitions and measurements of the outcome indicators were as follows:

Intervertebral disc protrusion (IDP):

The IDP was measured on the MRI axial position by drawing a line at the bottom of the disc and then making a vertical line to the point where the disc is most herniated.

Ratio value of disc grey scales (RVG):

The measurement of RVG was based on the modified Schneiderman method for assessing the water content of disc. The cerebrospinal fluid grey scale of sacral one segment was selected as the baseline reference value, and the grey scale of the responsible segmental disc was measured.

RVG = (average grayscale value of the responsible intervertebral disc / average grayscale value of cerebrospinal fluid sacral one segment) * 100%.
